# Supplementary material for: Crosslinking degree variations enable programming and controlling soft fracture via sideways cracking
Source: NPJ Comput Mater. 2024 Dec 16;10(1):282. doi: 10.1038/s41524-024-01489-y (PMC11649574; doi:10.1038/s41524-024-01489-y)
Supplement: Supplementary file 1 — Supplementary Material [file 41524_2024_1489_MOESM1_ESM.pdf]

SUPPLEMENTARY MATERIAL

Crosslinking degree variations enable programming and controlling soft fracture via sideways cracking

Miguel Angel Moreno-Mateos[a,*], Paul Steinmann[a,b]

[a] *Institute of Applied Mechanics, University of Erlangen–Nuremberg, Egerland Str. 5, 91058 Erlangen, Germany*
[b] *Glasgow Computational Engineering Centre, School of Engineering, University of Glasgow, G12 8QQ, UK*

* Corresponding author
Email address: `miguel.moreno@fau.de` (Miguel Angel Moreno-Mateos)

October 25, 2024

Supplementary Results 1: Rheological characterization of the curing process of Elastosil P7670

The rheological characterization of Elastosil P7670 with a mixing ratio of $\zeta = 1$ during curing was performed using a Discovery HR-30 rheometer (TA Instruments, New Castle, DE, USA) (Figure S1). A geometry with a diameter of $40\,\mathrm{mm}$ and a gap (height of the cylindrical sample) of $1000\,\mu\mathrm{m}$ were used. The angular velocity was $0.2\,\mathrm{rad\,s^{-1}}$ and the azimuthal shear strain, $1\,\%$. Note that the strain was measured at a radial coordinate of $r = \frac{2}{3}r_{\mathrm{max}}$, where $r_{\mathrm{max}} = 20\,\mathrm{mm}$. Curing the elastomer directly in contact with the geometry and Peltier plate ensured adhesion at the interfaces. The Peltier plate was maintained at a temperature of $25\,^{\circ}\mathrm{C}$ throughout the test.

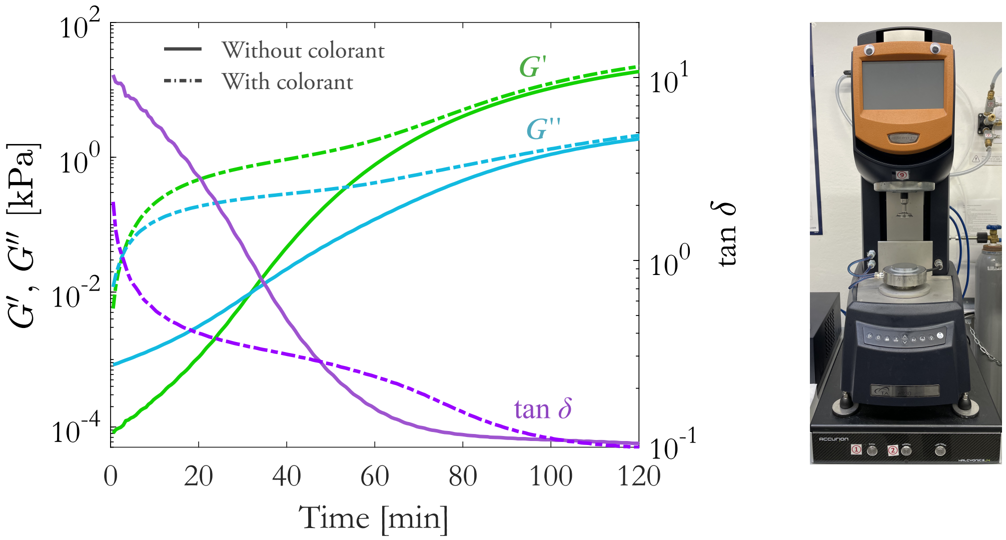

Figure S1. **Rheological characterization of Elastosil $\zeta = 1$ during curing at room temperature.** A shear rheometer measured the shear storage (G') and loss (G'') moduli of the elastomer at quasi-static deformation rate. Two tests were performed: one for the blend without colorant and one for the blend with colorant. The loss factor in shear mode is calculated as $\tan\delta = \frac{G''}{G'}$.

Supplementary Results 2: Tensile tests on uncut samples and calibration of the constitutive model

Tensile tests on uncut samples were utilized to calibrate the mechanical material parameters. To enhance the accuracy of this calibration, 3D simulations were conducted to replicate the actual boundary conditions of the specimens. In these simulations, the horizontal displacement at the upper and lower edges was constrained to zero, reflecting the clamping in the experimental setup. The mechanical contribution in the numerical model was calibrated using experimental results from tensile tests on virgin samples, as depicted in Figure S2.A. The parameters for the isochoric contribution, based on the Yeoh model, are listed in Table S1. The bulk modulus was chosen to be several orders of magnitude higher to guarantee near-incompressibility.

Table S1. Constitutive parameters of the Yeoh model used in the numerical simulations of the elastomer Elastosil P7670 manufactured according to the mixing ratios $\zeta \in \{0.5, 0.625, 0.75, 0.875, 1\}$.

ζ [-]	0.5	0.625	0.75	0.875	1
C_1 [kPa]	1.88	4.50	10.00	19.50	24.00
C_2 [kPa]	0.20	0.40	0.60	1.00	1.00
C_3 [kPa]	0	0	0.02	0.02	0.08

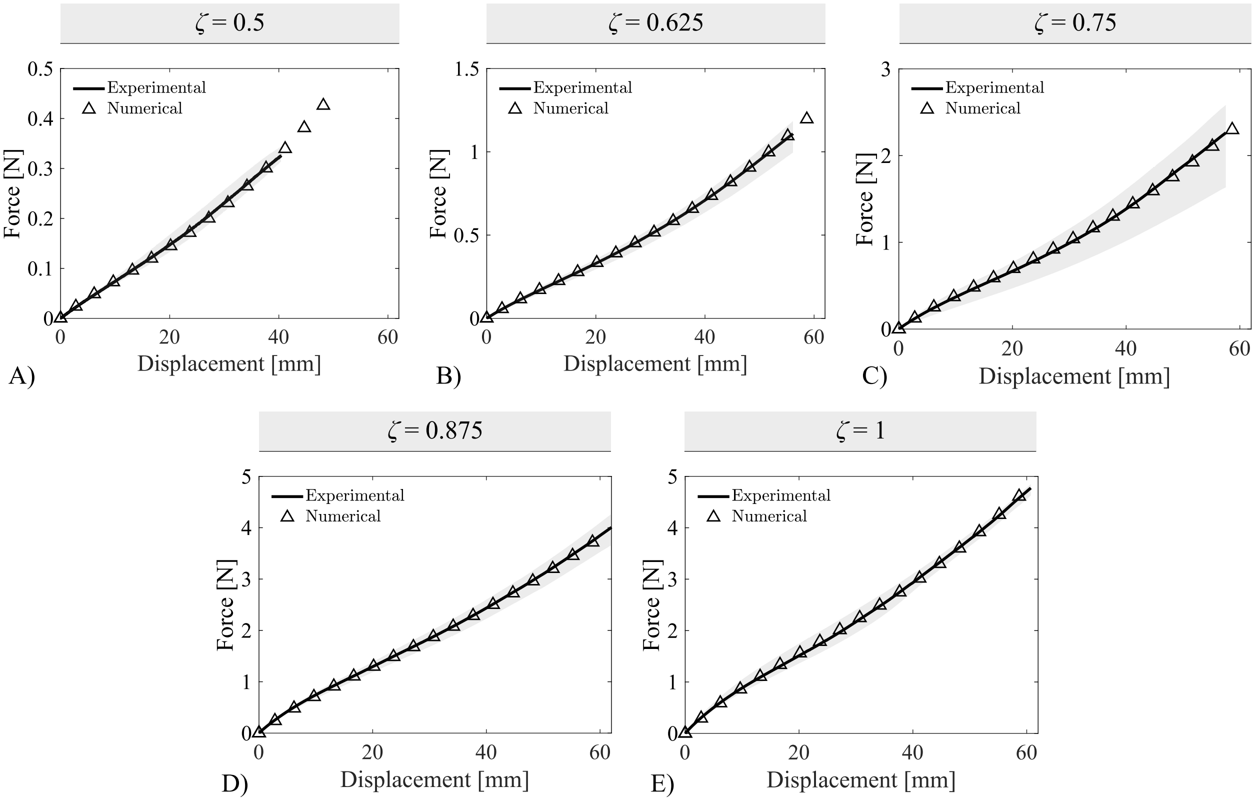

Figure S2. **Calibration of the Yeoh model from quasi-static tensile tests on uncut samples.** A numerical 3D finite element model reproduces the experimental setup to bridge the constitutive behavior with the force–displacement measurements obtained through the universal testing machine. The parameters of the Yeoh model, i.e., C_1, C_2, and C_3, are calibrated for samples produced with mixing ratios (ζ) of (A) 0.5, (B) 0.625, (C) 0.75, (D) 0.875, and (E) 1. For each ratio, the experimental mean curve is calculated from three experimental repetitions, whereby a scatter area is included to quantify the experimental variability.

Supplementary Results 3: Estimation of isotropic critical energy release rate

An estimation of the critical energy release rate ($G_{\text{c,iso}}$) for forward cracking was performed on samples with low crosslinking ($\zeta = 0.5$) according to the definition $G_{\text{c,iso}} = -\frac{\mathrm{d}U}{t\mathrm{d}c}$, as in [1, 2], with $\mathrm{d}U$ denoting the release of hyperelastic energy for a differential crack extension $\mathrm{d}U$ on a sample with a thickness t. Here, three estimations are made for finite crack extensions for a c/w from (A) 0.1 to 0.3, from (B) 0.3 to 0.5, and (C) from 0.1 to 0.5. Three similar values are obtained: $G_{\text{c,iso}} = 51.8 \times 10^{-3}\,\text{N}\,\text{mm}^{-1}$, $G_{\text{c,iso}} = 36.9 \times 10^{-3}\,\text{N}\,\text{mm}^{-1}$, and $G_{\text{c,iso}} = 38.5 \times 10^{-3}\,\text{N}\,\text{mm}^{-1}$, respectively. The average value $42.4 \times 10^{-3}\,\text{N}\,\text{mm}^{-1}$ is used. Figure S3 illustrates the results. In addition, Figure S3.D shows a comparison of the experimental and numerical fracture force-displacement curves. The displacement at failure in the numerical predictions is slightly higher than that in the experimental results. These deviations may be due to the regularization of the crack. Alternative strategies may include inverse parameter identification.

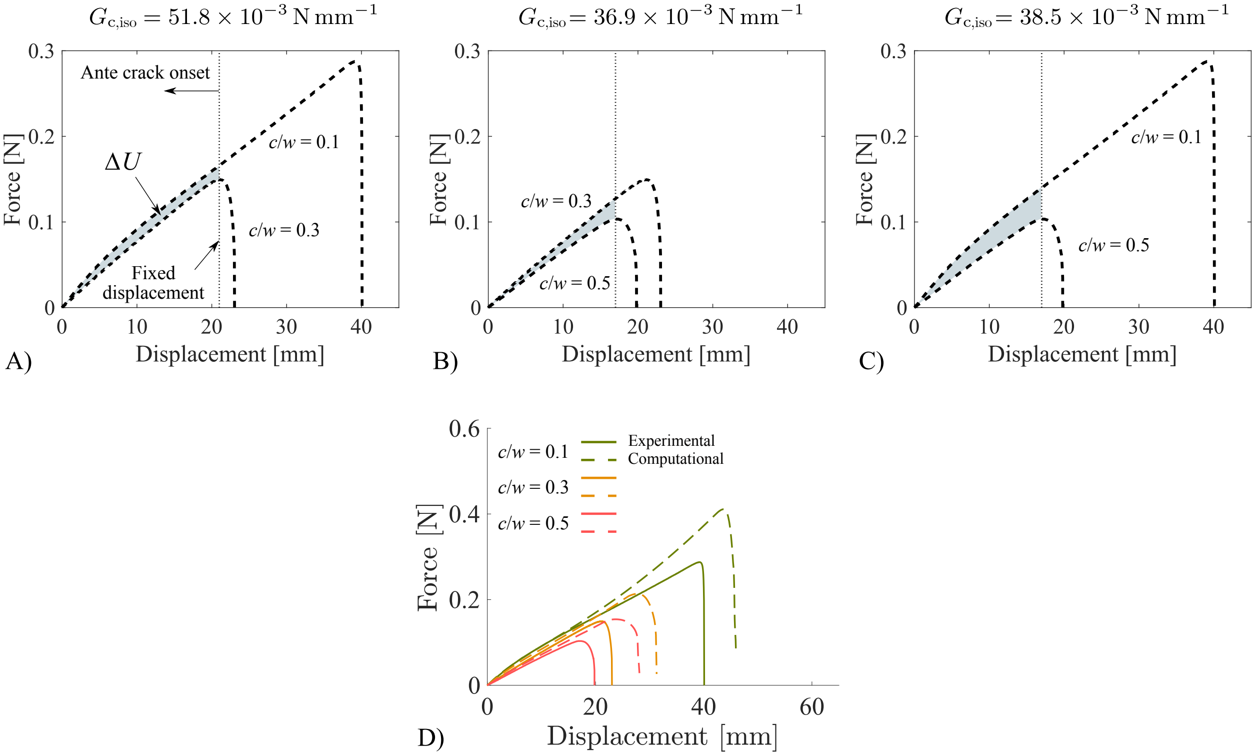

Figure S3. **Estimation of the isotropic critical energy release rate from samples with forward cracking.** Samples prepared for a mixing ratio (ζ) of 0.5 and low degree of crosslink enable estimations of the critical energy release rate as the difference of energy between force displacement curves on samples with pre-cuts of different lengths, defined through the crack width ratio (c/w), before crack onset. Three estimations are made for crack extension for a c/w from (A) 0.1 to 0.3, from (B) 0.3 to 0.5, and (C) from 0.1 to 0.5. Three similar values are obtained: $G_c = 51.8 \times 10^{-3}$ N mm^{-1}, $G_c = 36.9 \times 10^{-3}$ N mm^{-1}, and $G_c = 38.5 \times 10^{-3}$ N mm^{-1}, respectively. (D) Comparison of experimental and computational fracture force-displacement curves for the crack-width ratios.

Supplementary Results 4: Crack paths for samples with different crosslinking degrees and pre-cuts

Figures S4 and S5 present the results for crack extension in samples with mixing ratios of $\zeta \in \{0.625, 0.75, 0.875, 1\}$, which originate sideways cracking, in the material and spatial configurations, respectively. The data is shown for samples with pre-cuts of lengths $c \in \{1.2, 3.6, 6\}$ mm, corresponding to crack-width ratios of $c/w \in \{0.1, 0.3, 0.5\}$.

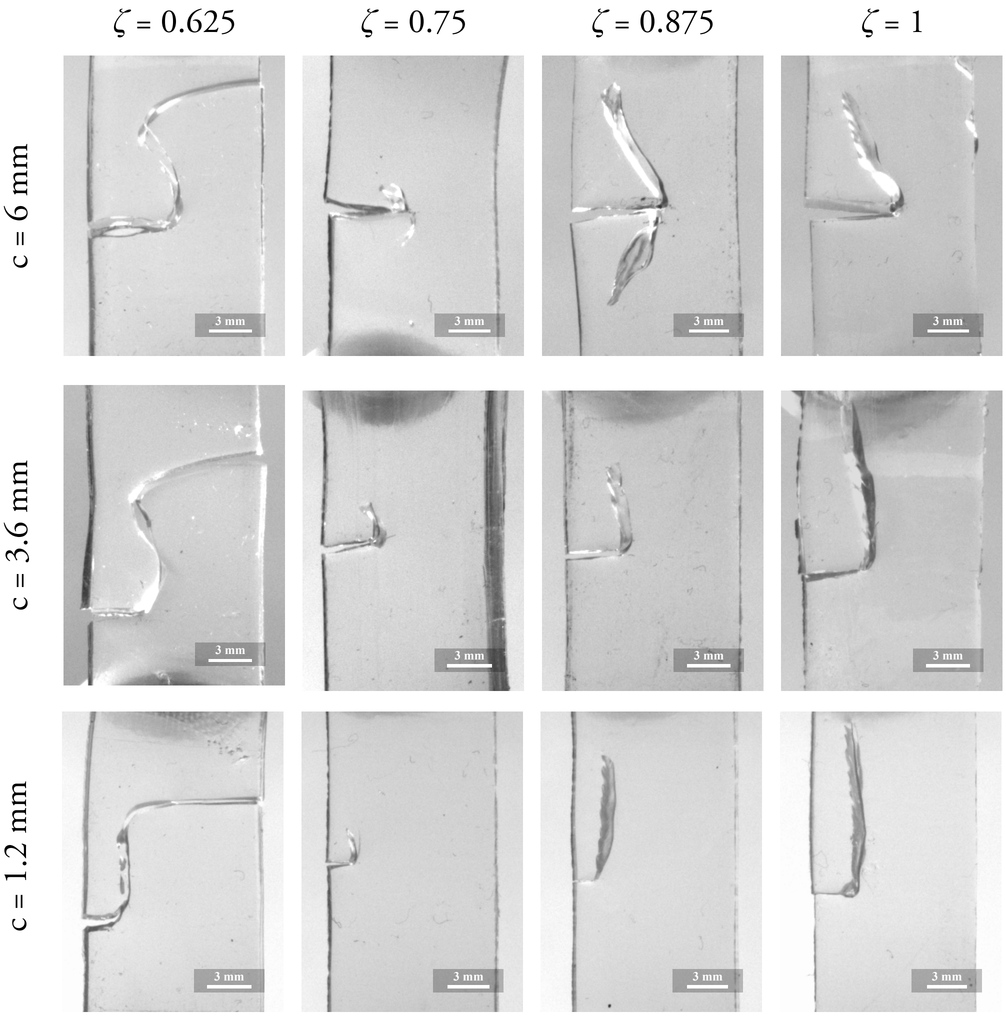

Figure S4. **Results for crack propagation for samples with different mixing ratios and initial pre-cuts in the undeformed configuration.** The images correspond to fully rupture for the low crosslinked sample with $\zeta = 0.625$ and to maximum sideways extension for $\zeta = 0.75$, 0.875, and 1. Low crosslinked samples for $\zeta = 0.5$ are not included since crack propagates forward and the path is the standard horizontal one.

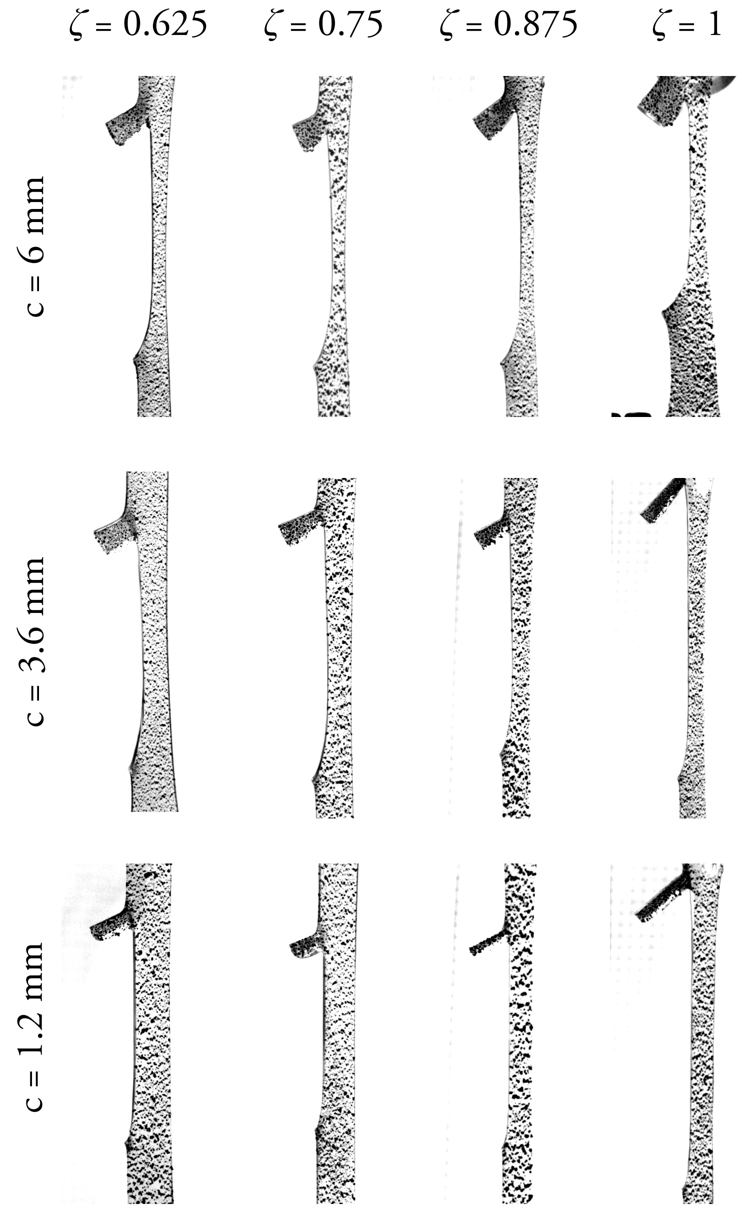

Figure S5. **Results for crack propagation for samples with different mixing ratios before full rupture and initial pre-cuts in the deformed configuration.** The results are presented for the mixing ratios of $\zeta = 0.625, 0.75, 0.875, 1$. The results for low crosslinked samples with $\zeta = 0.5$ are not included since crack propagates forward and the path is the standard horizontal one.

Supplementary Results 5: Computational results for anisotropic fracture with $\mathbf{m}_{\text{ani}}$ a non-local variable and parametric study in terms of ζ and $\tilde{\beta}_{\text{ani}}$

The vector $\mathbf{m}_{\text{ani}}$ is a non-local variable calculated from the principal (tensile) stretch direction as $\mathbf{m}_{\text{ani}} = \hat{\boldsymbol{\alpha}}_1 - l\,\nabla\hat{\boldsymbol{\alpha}}_1 \cdot \widehat{\nabla} d$, i.e., with $\hat{\boldsymbol{\alpha}}_1$ the principal eigenvector of the left Cauchy-Green tensor $\mathbf{b} = \mathbf{F} \cdot \mathbf{F}^{\text{T}}$. In turn, the normal chains direction in the material configuration, $\hat{\mathbf{M}}_{\text{ani}}$, is obtained through pull-back (contravariant) operation of the spatial counterpart, i.e., $\hat{\mathbf{M}}_{\text{ani}} = \mathbf{F}^{\text{-1}} \cdot [c\,\hat{\mathbf{m}}_{\text{ani}}]$, with c determined so that $\hat{\mathbf{M}}_{\text{ani}}$ is unit vector. Note that this is equivalent to the calculation of the principal eigenvector of the right Cauchy-Green tensor ($\mathbf{C} = \mathbf{F}^{\text{T}} \cdot \mathbf{F}$). In Fig. S6 (left) we illustrate the distribution of the material and spatial $\mathbf{M}_{\text{ani}}$ and $\mathbf{m}_{\text{ani}}$ vectors, respectively, with an example for the case $\zeta = 0.625$, $\tilde{\beta}_{\text{ani}} = 0.25$ at an intermediate load step, in material and spatial configurations.

The critical energy release rate is defined as a direction-dependent material function $G_{\text{c}} = G_{\text{c}}(\mathbf{F}, d)$ comprising isotropic ($G_{\text{c,iso}}$) and anisotropic ($G_{\text{c,ani}}$) contributions. The former ($G_{\text{c,iso}}$) represents the resistance to fracture in conventional forward cracking. The latter ($G_{\text{c,ani}}$) varies with the propagation direction, as it differs when propagating by cutting polymer chains compared to propagation parallel to the chains, and depends on the stretch in the direction of the chains. Consequently, the energetic requirements increase significantly for a crack propagating perpendicular to the chains aligned in the loading direction and experiencing severe stretching. In Fig. S6 (right) we illustrate the distribution of $G_{\text{c}} = G_{\text{c}}(\mathbf{F}, d)$ with an example for the case $\zeta = 0.625$, $\tilde{\beta}_{\text{ani}} = 0.25$ at an intermediate load step, in material and spatial configurations.

The propagation of sideways cracks in the computational model depends on the value of the anisotropy factor $\tilde{\beta}_{\text{ani}}$. The higher the value, the higher the resistant to forward propagation. To illustrate this dependency, Figure S7 shows the crack paths in the material (undeformed) configuration for the mixing ratios $\zeta \in \{0.625, 0.75, 0.875, 1\}$ and an array of values of $\tilde{\beta}_{\text{ani}}$. As the crosslinking degree increases, the amplification of the fracture anisotropy according to Equation 6 in the main manuscript enhances. We highlight the artifact in the top-right corner for $\zeta = 0.75$ and $\tilde{\beta}_{\text{ani}} = 0.4$ and for $\zeta = 0.875$ and $\tilde{\beta}_{\text{ani}} = 0.6$. The crack first propagates sideways, then bends forward, ultimately leading to full rupture. This is consistent with the crack pattern shown in Fig. S4.

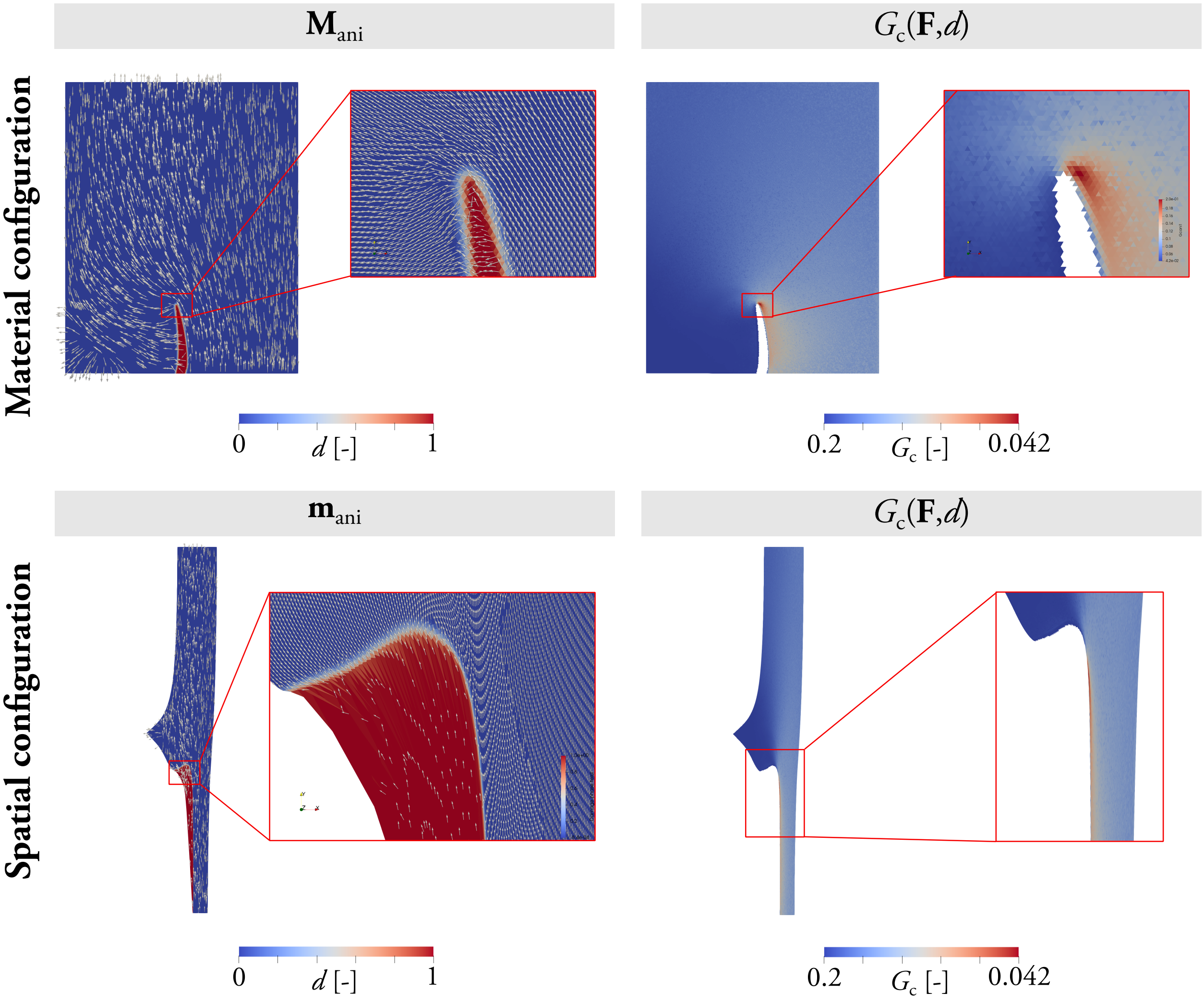

Figure S6. **Distribution of the material and spatial vectors $\mathbf{M}_{\text{ani}}$ and $\mathbf{m}_{\text{ani}}$, respectively, and critical energy release rate material function for a particular simulation with $\zeta = 0.625$ and $\tilde{\beta}_{\text{ani}} = 0.25$.** (Left) The vector $\mathbf{m}_{\text{ani}}$ is a non-local variable calculated from the principal (tensile) stretch direction as $\mathbf{m}_{\text{ani}} = \hat{\boldsymbol{\alpha}}_1 - l \, \nabla \hat{\boldsymbol{\alpha}}_1 \cdot \widehat{\nabla} d$, i.e., with $\hat{\boldsymbol{\alpha}}_1$ the principal eigenvector of the left Cauchy-Green tensor $\mathbf{b} = \mathbf{F} \cdot \mathbf{F}^{\text{T}}$. (Right) The critical energy release rate is a direction-dependent material function $G_c = G_c \left(\mathbf{F}, d\right)$.

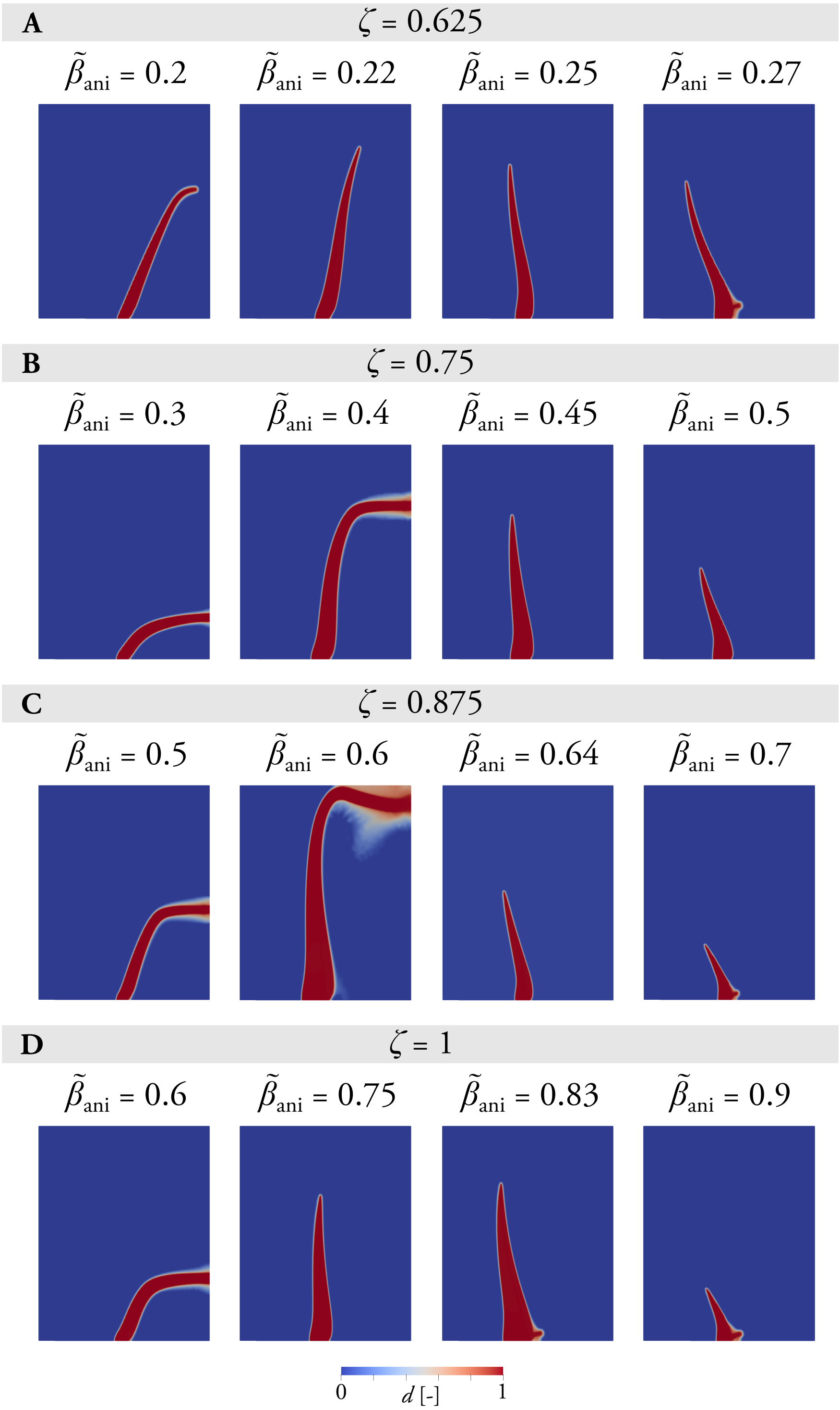

Figure S7. **Results for crack propagation for different parameters in the phase-field model and crack-width ratio** $c/w = 0.5$**.** The crack path is shown on the material (undeformed) configuration for sideways cracks produced for mixing ratios (ζ) of (A) 0.625, (B) 0.75, (C) 0.875, and (D) 1, and a variety of values of the anisotropic factor $\tilde{\beta}_{\text{ani}}$. The increase of the strain induced anisotropy occurs with the increase of this parameter. The constitutive behavior of the material varies with the mixing ratios according to the parameters in Table S1. The initial length of the pre-cut, modeled as a discontinuity in the mesh, is $6\,\text{mm}$ and the width of the samples (w), $12\,\text{mm}$.

Supplementary Results 6: Computational results for anisotropic fracture with $\mathbf{m}_{\text{ani}}$ a constant vector representing the loading direction in the experimental setup

In the sequel of Fig. S7 in the previous section, Fig. S8 shows the results from the simulations adopting $\mathbf{m}_{\text{ani}} = \{0, 1, 0\}$, i.e., a constant vector pointing in the vertical loading direction. This simplifies the critical energy release rate material function (Equation 4 in the main manuscript). We highlight the artifact in the top-right corner for $\zeta = 0.625$ and $\tilde{\beta}_{\text{ani}} = 0.2$. The crack first propagates sideways, then bends forward, ultimately leading to full rupture. This is consistent with the crack pattern shown in Fig. S4.

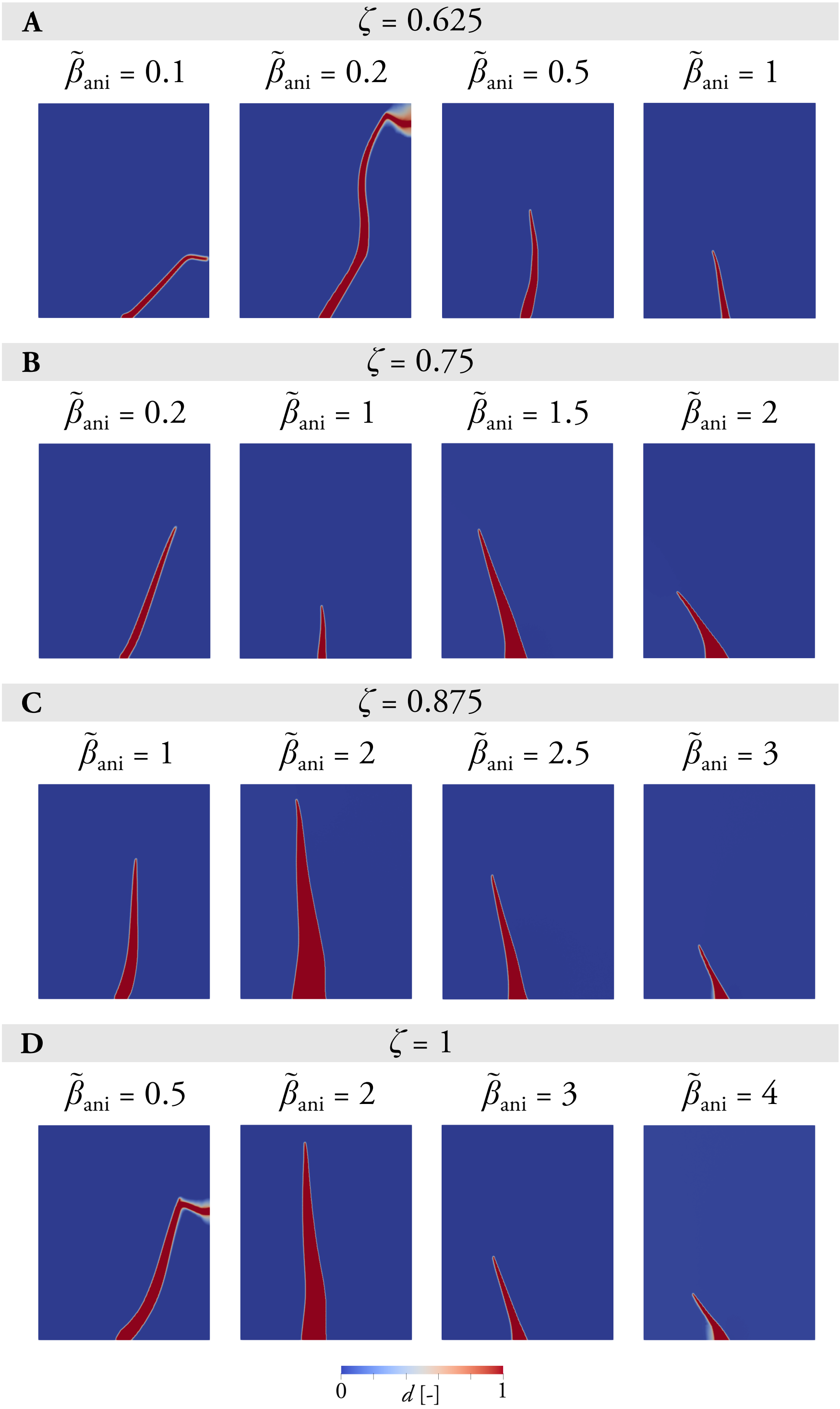

Figure S8. **Results for crack propagation for $\mathrm{m}_{\mathrm{ani}}$ a constant vector pointing in the vertical loading direction, different parameters in the phase-field model, and crack-width ratio $c/w = 0.5$.** The crack path is shown on the material (undeformed) configuration for sideways cracks produced for mixing ratios (ζ) of (A) 0.625, (B) 0.75, (C) 0.875, and (D) 1, and a variety of values of the anisotropic factor $\tilde{\beta}_{\mathrm{ani}}$. The increase of the strain induced anisotropy occurs with the increase of this parameter. The constitutive behavior of the material varies with the mixing ratios according to the parameters in Table S1. The initial length of the pre-cut, modeled as a discontinuity in the mesh, is $6\,\mathrm{mm}$ and the width of the samples (w), $12\,\mathrm{mm}$.

Supplementary Results 7: Computational results for plane-strain fracture of RVEs with high-crosslinked inclusions embedded in a low-crosslinked soft matrix

Figure S9 showcases the results for the 3D-simulations on Representative Volume Elements (RVE) with inclusions and reinforcements under alternative boundary conditions: z-displacement fixed to zero on the $x - y$ plane on the side of the RVE.

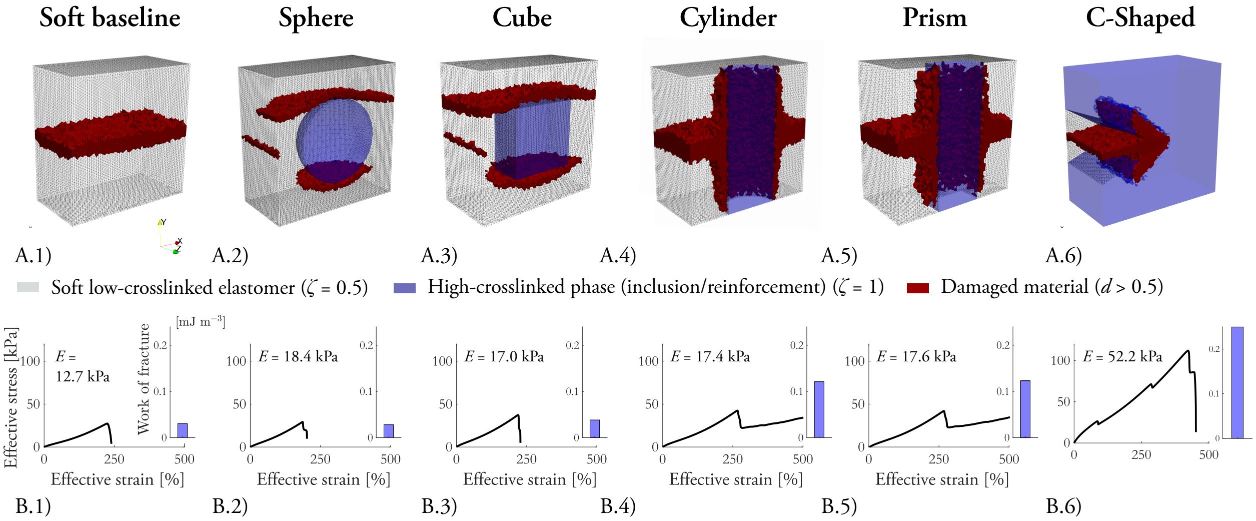

Figure S9. **Results for the fracture behavior of Representative Volume Elements with high-crosslinked inclusions ($\zeta = 1$) embedded in a low-crosslinked soft matrix ($\zeta = 0.5$).** The z- displacement is fixed to zero on the lateral sides ($x - y$ symmetry plane and parallel plane on the side of the RVE) and x-displacement is not constrained. Initial damage ($d = 1$) is prescribed on one side at the middle of the height. Computational domain in the material (undeformed) configuration where fracture has evolved ($d > 0.5$) for a (A.2) spherical inclusion, (A.3) cylindrical fiber, (A.4) cubic inclusion, (A.5) prismatic fiber, and (A.6) C-shaped inclusion. (A.1) A purely low-crosslinked RVE is included as a baseline for comparison to the other cases. The length of the edges of the cubic RVEs is $10.00\,\mathrm{mm}$, the radius of the centered spherical inclusion $3.00\,\mathrm{mm}$, $1.90\,\mathrm{mm}$ radius for the cylindrical fiber, the edge of the cubic inclusion $4.84\,\mathrm{mm}$, and $3.36\,\mathrm{mm}$ for the prismatic fiber. These four RVEs maintain the same inclusion volume ratio of $11.3\,\%$. (B.1-6) Curves with the engineering average stress against the engineering average strain for all cases, including the work of fracture as the area under the curves divided by the total volume of the RVE. For the Cylinder and Prism fiber cases, the work of fracture is calculated up to a displacement of $50\,\mathrm{mm}$. The apparent Young's modulus is calculated from the engineering stress-strain curves.

Supplementary Video 1: Animation of the deformation and fracture propagation in a C-Shaped RVE obtained with the computational model

References

[1] Moreno-Mateos, M. A., Hossain, M., Steinmann, P. & Garcia-Gonzalez, D. Hard magnetics in ultra-soft magnetorheological elastomers enhance fracture toughness and delay crack propagation. *Journal of the Mechanics and Physics of Solids* **173**, 105232 (2023).

[2] Moreno-Mateos, M. A., Mehnert, M. & Steinmann, P. Electro-mechanical actuation modulates fracture performance of soft dielectric elastomers. *International Journal of Engineering Science* **195**, 104008 (2024).
